# Dual Potential of Cetuximab Conjugated Hydroxyapatite Zirconium Nanoparticle as Nanocarrier for Radioenhancer in X-Ray Dynamic Therapy and ^177^Lu-based Radioimmunotherapy of Lung Cancer

**DOI:** 10.7150/ntno.101699

**Published:** 2025-03-03

**Authors:** Ahmad Kurniawan, Isa Mahendra, Asep Rizaludin, Marhendra Satria Utama, Ronny Lesmana, Fitria Dwi Ayuningtyas, Maria Rosa Dewanti, Dani Gustaman Syarif, Muhamad Basit Febrian

**Affiliations:** 1 Research Center for Radioisotope, Radiopharmaceuticals and Biodosimetry Technology, National Research and Innovation Agency, Serpong, Indonesia.; 2 Department of Radiology, Faculty of Medicine, Universitas Padjadjaran/Dr. Hasan Sadikin General Hospital, Bandung, Indonesia.; 3 Department of Biomedical Science, Physiology Division, Faculty of Medicine, Universitas Padjadjaran, Bandung, Indonesia.; 4 ITB Olympus Bioimaging Center, Bandung Institute of Technology, Bandung, Indonesia.; 5 Indonesian Polytechnic of Nuclear Technology, Sleman, Indonesia.; 6 Research Center for Radiation Process Technology, National Research and Innovation Agency, Serpong, Indonesia.

**Keywords:** Hydroxyapatite, Cetuximab, Nanoparticle, X-ray dynamic therapy, Radioimmunotherapy, Lung cancer

## Abstract

This study aimed to synthesize cetuximab (CTX) conjugated hydroxyapatite zirconium (HApZr-CTX) as a nanocarrier for active delivery of photosensitizer and therapeutic radionuclide. The system enabled targeted radioenhancer in X-ray dynamic therapy and radioimmunotherapy for lung cancer. The results showed that HApZr-CTX had the main characteristics of hydroxyapatite crystal in X-ray powder diffraction (XRD), with particle size twice bigger, according to DLS-PSA and TEM measurements. Cellular ROS generation was elevated almost three times in A549 cells after being treated using 125 µg/mL HApZr-CTX and irradiated with 5 Gy of X-ray photon compared to untreated cells. The viability of the treated lung cancer cell line decreased after exposure to external radiation. Moreover, as a radioimmunotherapy candidate, ^177^Lu was successfully loaded into HApZr-CTX nanocarrier and internalized in A549 more than half of the given dose after 0.5 h incubation. [^177^Lu]Lu-HApZr-CTX was primarily accumulated in the lung organs of healthy mice one-hour post-injection. In conclusion, HApZr-CTX nanoparticles have the potential to be used as a radioenhancer in X-ray dynamic therapy and radioimmunotherapy for lung cancer therapy.

## Introduction

Targeted or personalized therapy is the next-generation method for cancer treatment using carriers, such as nanoparticles, to deliver anticancer drugs to the specific target receptor. Nanoparticles can be engineered to target specific cells or tissues in the body by modifying the surface with molecules. This carrier improves drug delivery and reduces side effects by preventing healthy cells uptake of loaded drugs, including photosensitizer and β-emitting radionuclides, as well as mitigates the adverse effects in healthy cells 1.

Lung cancer reportedly has the highest fatality rate in the world, the first among men and second leading among women 2. The development of methods for diagnosing and treating cancer is required to increase the effectiveness of therapy and minimize side effects. The ultimate objective is to increase the survival rate and quality of life for cancer patients. Several studies are presently in search of novel methods apart from standard therapy, such as chemoradiotherapy and surgery 3.

One of the nanoparticles known to treat cancer cells is hydroxyapatite. Aside from the anticancer activity, hydroxyapatite nanoparticles (HApNPs) also have unique properties as drug carriers 4. HApNPs could passively accumulate at cancer sites with enhanced permeability and retention effect. However, accumulation in healthy tissues hampers the effectiveness and safety 5. Modifying HApNPs surface with targeting molecules can strengthen its selectivity against specific cancer receptors, allowing the therapy mechanism to be focused and reducing side effects.

EGFR (epidermal growth factor receptor) mutation is common in lung cancer, particularly among young people, women, and those of Asian descent. This mutation is mainly observed among patients with adenocarcinoma, non-smokers, and Eastern Asian ethnicity. Among Asian patients, the overall prevalence of EGFR mutations is 50% or higher. EGFR sensitizing mutation is the most common driver gene in non-smoker adenocarcinoma, constituting 60-78% of this subgroup 6-8. Therefore, targeted therapy with EGFR as a target receptor could be crucial in treating lung adenocarcinoma in Asians.

Targeting molecules, also known as ligands, can be added to the surface of NP to guide delivery to the specific receptors of the cells. When EGFR acts as a receptor for targeted therapy, the molecules would be anti-EGFR. Meanwhile, cetuximab (CTX) is a monoclonal antibody that binds to EGFR and inhibits activation. It interacts exclusively with domain III of the soluble extracellular region, partially occluding the ligand binding region on this domain and sterically preventing the receptor from adopting the extended conformation required for dimerization 9. CTX has been studied as a therapy for non-small cell lung cancer (NSCLC). In a phase II clinical trial, adding CTX to cisplatin plus vinorelbine as first-line therapy resulted in promising efficacy. Patients treated with CTX had higher response rates (35% versus 28%) and longer progression-free survival (hazard ratio 0.71, median 5.0 versus 4.6 months). More recently, a clinical trial evaluated the combination of CTX and nivolumab, an anti-PD-L1 drug, as second or third-line therapy in NSCLC patients. Clinically relevant anti-tumor activity was observed in 6 out of 16 patients, with progression-free survival of 8 months or more 10-12.

This study investigated the synthesis of CTX-modified HApNPs as a drug delivery system for ^177^Lu, a β-emitting radionuclide, in radioimmunotherapy of lung cancer. Combining NP with antibodies can maintain optimal active drug concentration and minimize systemic toxicity 13. Therefore, the conjugate of NP and monoclonal antibodies is expected to accelerate the uptake of ^177^Lu into specific tumor sites compared to conventional radioimmunotherapy using radiolabeled monoclonal antibodies 14,15. The long circulation time of radionuclide-labeled antibodies has been a significant shortcoming of radioimmunotherapy. Molecules with molecular weights above 70 kDa, the glomerular filtration rate threshold, remain in circulation longer compared to smaller, more rapidly eliminated ones 16. This long circulation time is beneficial in some cases, but may also result in radiation exposure to healthy tissues and organs, which can cause side effects 17.

Aside from being designated for radioimmunotherapy, the nanomaterial in this study has the potential to be used in X-ray dynamic therapy (XDT), a promising alternative to cancer therapy. The trend of XDT studies that offer improvements to photodynamic therapy has recently increased. Using X-rays instead of visible rays potentially improves the effectiveness of PDT because X-rays can penetrate body tissues and be used for deep-seated tumours. Metallic nanoparticles possess radiosensitizing properties. When exposed to X-ray radiation, these nanoparticles generate Auger electrons, Compton electrons, secondary electrons, and photoelectrons, facilitating the breakdown of water and oxygen molecules into free radicals. This process significantly increases the production of ROS within cells. Such nanoparticles, known as radioenhancers, hold promise for enhancing the efficacy of radiotherapy 18,19. This property differs between XDT and X-ray photodynamic therapy (X-PDT), where X-ray is converted into a photon by a radiosensitizer, and then the photosensitizer converts this photon into ROS. Chen successfully incorporated hafnium as a radio enhancer into the HAp crystal, and the material showed anticancer activity through the generation of reactive oxygen species (ROS) after irradiation of HAp-Hf with X-ray. 20. A previous study evaluated the anticancer potential of zirconium doping into HAp instead of hafnium, although the efficacy of XDT has yet to be investigated 5.

CTX conjugated hydroxyapatite is yet to be investigated as a nanocarrier platform for radioenhancer and therapeutic radionuclide delivery. Therefore, this study aimed to evaluate whether zirconium-doped HApNPs surface modified with CTX could generate intracellular reactive oxygen species (ROS) following X-ray irradiation and specifically deliver cytotoxic β-radiation from ¹⁷⁷Lu to EGFR-expressing cancer cell lines, with targeted accumulation in lung tissue.

## Methods

### Synthesis of Hydroxyapatite Zirconium (HApZr) Nanoparticle

HApZr nanoparticle was synthesized using the hydrothermal method 20. Solution A was prepared by dissolving CaCl_2_.2H_2_O and Zr (NO_3_)_4_ with a molar ratio of 85:15 and a total concentration of 0.2 M in distilled water. Meanwhile, solution B was prepared by dissolving (NH_4_)_2_HPO_4_ using distilled water with a P molar ratio following the (Ca+Zr)/P formula equal to 1.67. Solution B was added dropwise into solution A, and pH was adjusted to 8 with NH_4_OH. The mixture was stirred vigorously for 2 h at 80°C and left for 24 h. The synthesized suspension was purified from chloride ions and other impurities by washing with distilled water and ethanol, then dried using a freeze dryer for 72 h.

### Cetuximab modified HApZr (HApZr-CTX)

About 0.05 g HApZr was added with 2.5% v/v (3-Aminopropyl)triethoxysilane (APTES) in 5 mL toluene or ethanol, then stirred for 3 h at room temperature. Subsequently, the mixture was centrifuged and rinsed with ethanol three times. Solids were analyzed by FTIR (Fourier transform infrared) and DLS PSA, then 10 mg HApZr-APTES was dispersed in 5 mL deionized water and ultrasonicated for 30 minutes. About 0.5 mL HApZr-APTES dispersion was pipetted into a 1.5 mL microtube and 250 µL each of 0.4M EDC and 0.1M NHS was added into the microtube before the addition of 100 µg CTX and incubation at 25°C for various time. The solution was rinsed 3x with water, centrifuged at 10,000 rpm for 5 minutes, and freeze-dried. Once optimum conjugation time was achieved, the loading capacity of CTX onto HApZr-APTES surface was determined by varying the concentration.

### Quantification of Cetuximab Conjugated to HApZr

CTX conjugated to HApZr was quantified by measuring the amount remaining in the supernatant after conjugation 21. A modified Bradford test using Bio-Rad® protein assay kit I 5000001 was used to determine the amount of CTX residue 22. CTX standard solutions (0 - 50 ug/mL) were prepared in a 2 mM phosphate buffer (pH 8.0). HApZr-CTX conjugates, and the standard solutions were centrifuged at 10,000 rpm for 10 minutes. The supernatant was collected and aquabidest as a wash solution was added before resuspension of HApZr-CTX. This washing step was carried out three times and 90 µL of supernatant, wash solution, or standard was poured into a 96-well plate. Each sample was diluted with 70 µL of 2 mM PBS buffer before adding 40 µL of the Bio-Rad reagent. Incubation was carried out at room temperature for 15 minutes.

The microassay procedure was conducted to measure the lower concentration of CTX (0.07 g/mL). This procedure requires 200 µL of Bio-Rad reagent added to 800 µL of sample or standard solution. Subsequently, absorbance was measured in a 1 cm sample cell using UV-Vis spectrophotometer (Thermo Scientific Genesys 30). The overall amount of CTX molecules adsorbed onto HApZr nanoparticle was calculated by subtracting the total CTX added to the suspension with measured residual CTX.

### Material characterization

Bruker D8 Advance X-ray powder diffraction (XRD) system equipped with CuKα radiation (λ = 1.541874 Å) generated at 40 kV with a 40-mA current and recorded with a Lynx-eye sensitive detector was used to characterize the diffractogram patterns of HApZr nanoparticle. Nanoparticle was scanned with a 0.02^°^ step size between 8 and 60°C. Using the Profex program, the patterns were examined and compared to the diffraction data obtained from the International Center for Diffraction Data (ICDD). The Scherrer equation from (002) and (310) reflection at 2θ = 26◦ and 2θ = 39◦ diffraction peaks, respectively, was used to compute the lattice parameters (c-axis and a-axis). Furthermore, FTIR analysis was used to identify the functional groups present on the surface of nanoparticle. This analysis would capture the middle infra-red range (MIR) number between 400 and 4000 cm^-1^ at ambient temperature using Bruker ® Alpha II Compact FTIR Spectrometer. The particle size distribution of the HApZr-APTES and HApZr-CTX was analyzed using the HORIBA LB-550 Dynamic Light Scattering Particle Size Analyzer (DLS-PSA). Both nanoparticle were dispersed and diluted using deionized water before measurement. The size and morphology of nanoparticle were analyzed using Transmission Electron Microscopy (TEM; JEM-1400 Plus) (JEOL USA, Peabody, Massachusetts).

### Radiolabeling with ^177^Lu

About 100 µL of 1 mg/mL HApZr-CTX dispersion was pipetted into microtubes before the addition of aquabidest to reach 500 µL followed by homogenization by gentle shaking. Subsequently, 74 MBq of [^177^Lu]LuCl_3_ (LutaPol® Polatom, specific activity 557 GBq/mg Lu, radionuclidic purity 99.9%, radiochemical purity 99.5%) in 0.5M ammonium acetate (2:1 v/v) was added to HApZr-CTX solution. The mixture was then incubated at room temperature for 30 minutes with gentle shaking.

The radiochemical purity and yield of [^177^Lu]Lu-HApZr-CTX were determined by chromatography on Whatman-31ET paper using 5 mM diethylenetriaminepentaacetic acid (DTPA) as the mobile phase. Retention factor (Rf) of the radiolabeled HApZr-CTX was 0-0.1, while free ^177^Lu migrated to the solvent front in Rf 0.8-0.9.

### Cellular uptake study of [^177^Lu]Lu-HApZr-CTX

The human lung cancer A549 was obtained from the American Type Culture Collection (ATCC). The cells were then preincubated into 24 well plates overnight with a density of 1x10^6^ cells/well. The culture medium was removed, and the cells were washed using HBSS three times, then[^177^Lu]LuCl_3_ and [^177^Lu]Lu-HApZr-CTX (radioactivity 1.25 MBq/100 µL) were added into each well and incubated for 0.25 h, 0.5 h, and 1 h. After incubation, the cells were washed using HBSS three times and lysed using 0.2 M NaOH (Supelco). The cell lysate was collected into a polystyrene tube and counted using the Automatic Gamma Counter (2470 Wizard2™, PerkinElmer).

### Fluorescence labeling of HApZr-CTX

About 0.2 mg of HApZr-CTX (1 mg/mL) was pipetted into a microtube and 20 μL FITC (1 mg/mL in DMSO) was added. The solution was stirred for 2 h at room temperature, centrifuged, rinsed three times with deionized water, and then reconstituted in 1 mL of deionized water.

### Cellular imaging of HApZr-CTX

Cellular imaging was analyzed using a Confocal Laser Scanning Microscope (CLSM FV1200, Olympus, Japan) in the ITB-Olympus Bioimaging Center (IOBC). After 24 hours of incubation, the internalization of HApZr-CTX conjugated with FITC into the culture of A549 cancer cells was observed. Nuclei staining by 4′,6-diamidino-2-phenylindole (DAPI) was observed with a 405 nm laser and FITC using a 488 nm laser in the CLSM instrument.

### Determination of cellular ROS

ROS generation was evaluated using 2'7'-Dichlorofluorescin diacetate (DCFH-DA) (Sigma Aldrich, Cat. No. D6883). The A549 cell line was then cultured into 96 well plates with a density of 1x10^4^ cells/well using DMEM (Sigma Aldrich, Cat. No. D5796) supplemented with 10% Fetal Bovine Serum (FBS) (Sigma Aldrich, Cat. No. F2442) and 1% Penicillin Streptomycin (Gibco, Cat. No.15140122). The cells were maintained using a CO_2_ incubator for 24 h, then treated using HApZr-CTX with a variant dose and H_2_O_2_ as a positive control. After incubation, the cells were irradiated using a Varian Clinac iX 6MV photon beam with a 5 Gy dose. DCFH-DA was added and the solution was incubated for 40 minutes. The cells were washed using Hanks Balanced Salt Solution (HBSS) (Gibco, Cat. No. 14025092). ROS generation was measured using a TECAN fluorescence microplate reader with 485 nm excitation and 535 nm emission.

### Cytotoxic studies of X-ray dynamic therapy

Cytotoxic studies using XDT were evaluated and assessed with the Cell Proliferation Reagent WST-1 (Roche Diagnostics GmbH, Mannheim, Germany). The A549 cell line was cultured in a 96-well plate at a density of 1 × 10^4^ cells/well and incubated overnight at 37°C with 5% CO_2_. The cells were treated with HApZr-CTX at varying concentrations of 100, 25, 12.5, 6.25, 3.125, 1.563, and 0.781 μg/mL 1 h before irradiation. Following this, the cells were irradiated using a Varian Clinac iX 6mv photon beam with a 5 Gy dose, then incubated for 24 h and rinsed with a complete medium. About 10 μL of the WST-1 reagent was added into each well containing 100 μL of complete medium. Before measuring the absorbance, cells were incubated for 3 h. The absorbance was measured using a microplate reader at 480 nm. The IC_50_ of HApZr-CTX after irradiation was then determined and compared to the non-irradiated control group.

### Animal study

All the experimental procedures in this study have been approved by the Institutional Animal Care and Use Committee of the National Research and Innovation Agency of Indonesia (No. 010/KE.02/SK/6/2022).

### Biodistribution study

[^177^Lu]Lu-HApZr-CTX (1.25 MBq/100 µL) was injected intravenously into the tail vein of BALB/c mice (n = 3). Mice were euthanized 1 h after injection based on the accepted protocol. The organ of interest was collected, and the activity was measured using an Automatic Gamma Counter with NaI(Tl) detector. [^177^Lu]Lu-HApZr-CTX biodistribution was expressed as the percentage of injected activity dose per organ weight (%ID/g).

### Statistical analysis

Statistical evaluation was conducted using GraphPad Prism (La Jolla, CA, USA, version 10.1.0) and data were presented as mean ± SD. Student t-test was used to determine significant differences between groups and *p-value* less than 0.05 was considered statistically significant.

## Results

### Synthesis of Hydroxyapatite Zirconium (HApZr) nanoparticle

**Figure 1:**
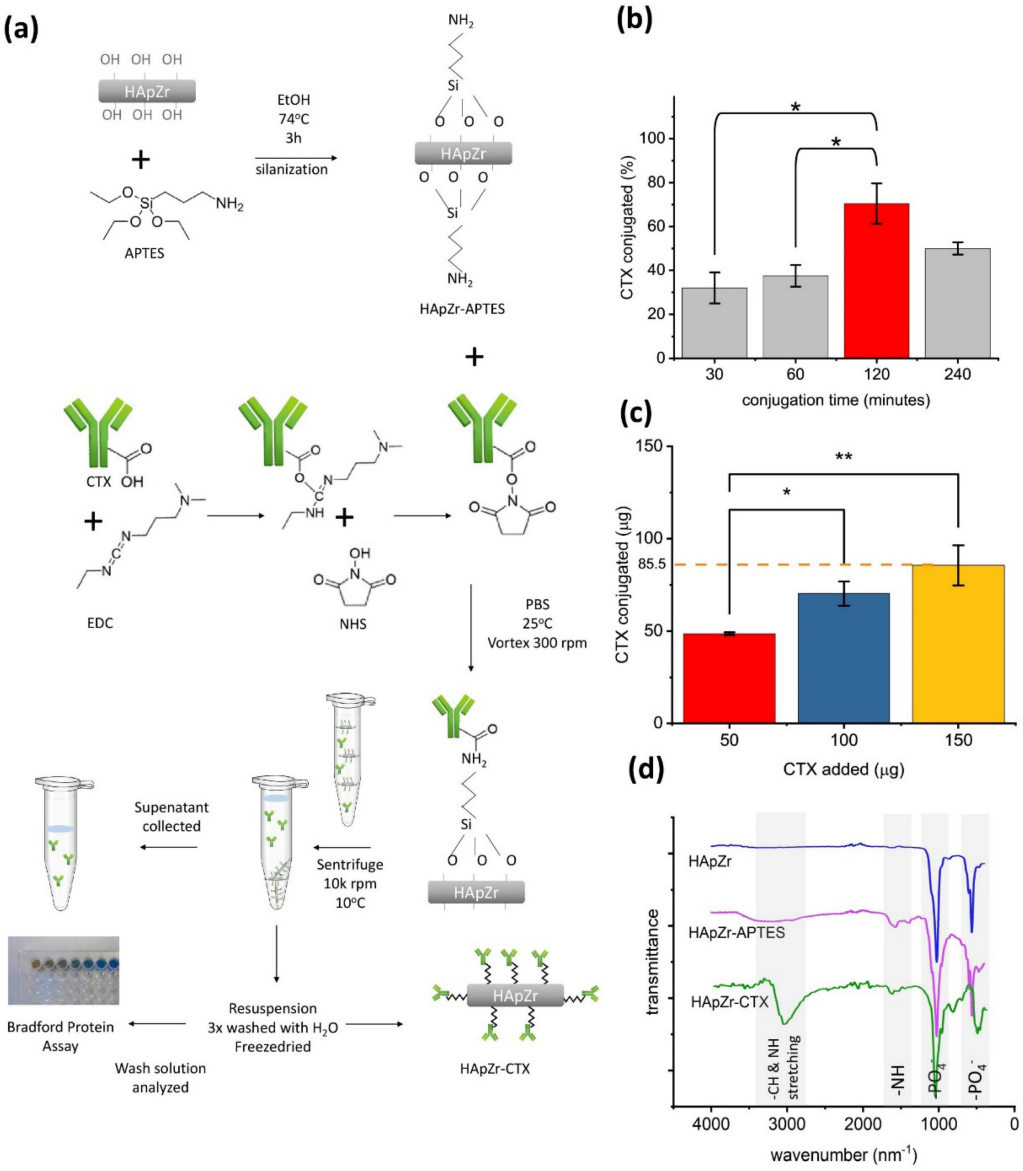
(a) Scheme of surface modification of HApZr using CTX (b) Time optimization for CTX conjugation onto HApZr, 200 µg HApZr-APTES, 100 µg CTX, vortex mixer 300 rpm, 25°C (c) Loading capacity of CTX onto HApZr-APTES, 200 µg HApZr-APTES, 120 minutes reaction, vortex mixer 300 rpm, 25°C (d) FTIR spectra of HApZr, HApZr-APTES and HApZr-CTX.

HApZr nanoparticle was synthesized based on a previous study 5,23. A slight modification was applied using the hydrothermal method rather than the cold, wet chemical precipitation method because it is faster and yields properties similar to previous studies. Modification of HApZr surface with amine group was performed before conjugation with CTX as a specific directing agent for tumors with EGFR overexpression. The amine groups was attached through APTES by reaction in an alcoholic solvent. APTES and EDC-NHS coupling method is widely used to synthesize antibody nanoparticle conjugates. These method create a stable amide-silane bond for anchoring monoclonal antibodies to the surface of nanoparticle. Figure 1.a shows a schematic diagram of HApZr surface modification with CTX and characterization. The results showed that optimum conditions for conjugation reaction was 120 minutes reaction in room temperature with gentle shaking (Figure 1.b). Furthermore, the maximum loading capacity of CTX in HApZr surface was found to be 85.5 ± 10.8 µg CTX/1 mg HApZr according to the linear fitting equation shown in Figure 1.c. pH during the conjugation reaction was 6, following the value for deionized water as a dispersant of nanoparticle.

FTIR graph in Figure 1.d shows the vibration of the main group in HApZr nanoparticle, including P-O vibration at 1075 and 1052 cm^-1^. NH_2_ vibration at 1548 cm^-1^ was detected after modification of HApZr surface with APTES. This peak shifted into amide I vibration at 1620 cm^-1^ after CTX addition and EDC/NHS. C-H stretching from CTX protein was also detected as a broadening peak in 2980 cm^-1^. This amide peak and C-H stretching indicated that HApZr surface was successfully modified with CTX. Furthermore, Bradford protein test confirmed the attachment of CTX into HApZr surface. The residual CTX, which did not attach to HApZr, remained at the supernatant after high-speed centrifugation.

**Figure 2:**
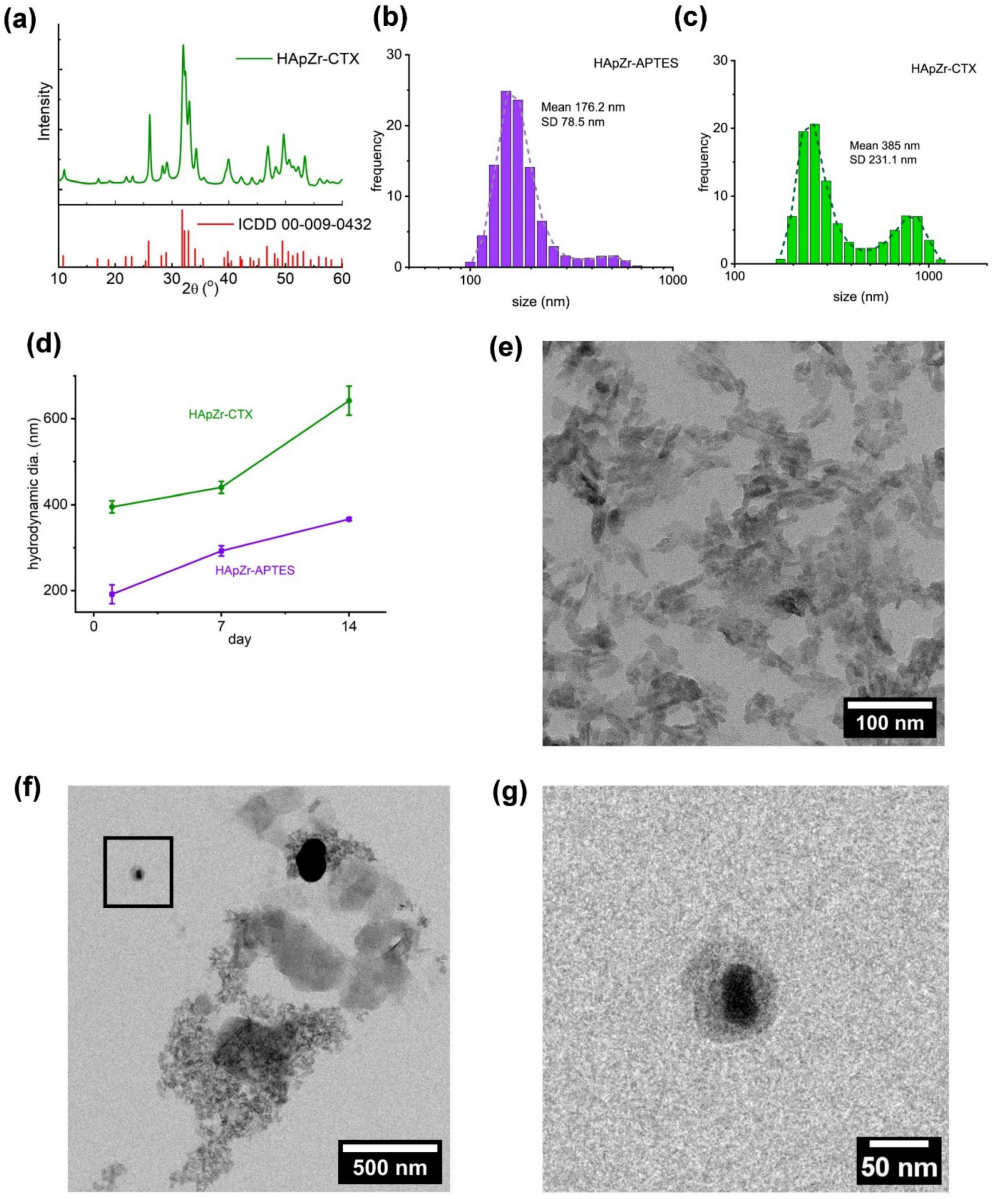
(a) XRD diffractogram of HApZr-CTX compared to ICDD 00-009-0432 of standard hydroxyapatite, (b) DLS-PSA analysis of HApZr-APTES and (c) HApZr-CTX dispersion in water, (d) hydrodynamic size stability of HApZr-APTES and HApZr-CTX dispersion in water for 14 days in 10°C storage, (e) TEM image of bare HApZr nanoparticle showed bar type nanoparticle with average size of 33 nm, (f) TEM image of agglomerated HApZr-CTX nanoparticle with size around 500 nm, note that thick shadow was alleged as CTX bound to agglomerated HApZr nanoparticle, (g) enlarged inset of HApZr-CTX nanoparticle from figure 2.f showed smaller HApZr-CTX nanoparticle with particle size around 50 nm

Material characterization of HApZr-CTX showed that HApZr crystal was nearly identical to standard hydroxyapatite according to XRD analysis as shown in Figure 2.a. HApZr and HApZr-CTX have similar crystal size based on TEM micrograph but show differences in the morphology of shadow around crystal due to addition of APTES and CTX. Figure 2.g shows APTES and CTX shadow encircling the bold HApZr crystal. This shadow was absent in the bare HApZr micrograph, as depicted in Figure 2.e. APTES and CTX layers were bound to several clusters of HApZr nanoparticle, leading to the agglomeration. This agglomeration caused a bigger hydrodynamic size, as shown in Figure 2.c and 2.f, compared to HApZr-APTES size in Figure 2.b. HApZr-APTES hydrodynamic size grew slowly during 14 days of storage at 10°C, but the growth was more progressive in HApZr-CTX as seen in Fig 2.d.

### Loading of ^177^Lu into HApZr-CTX

**Figure 3:**
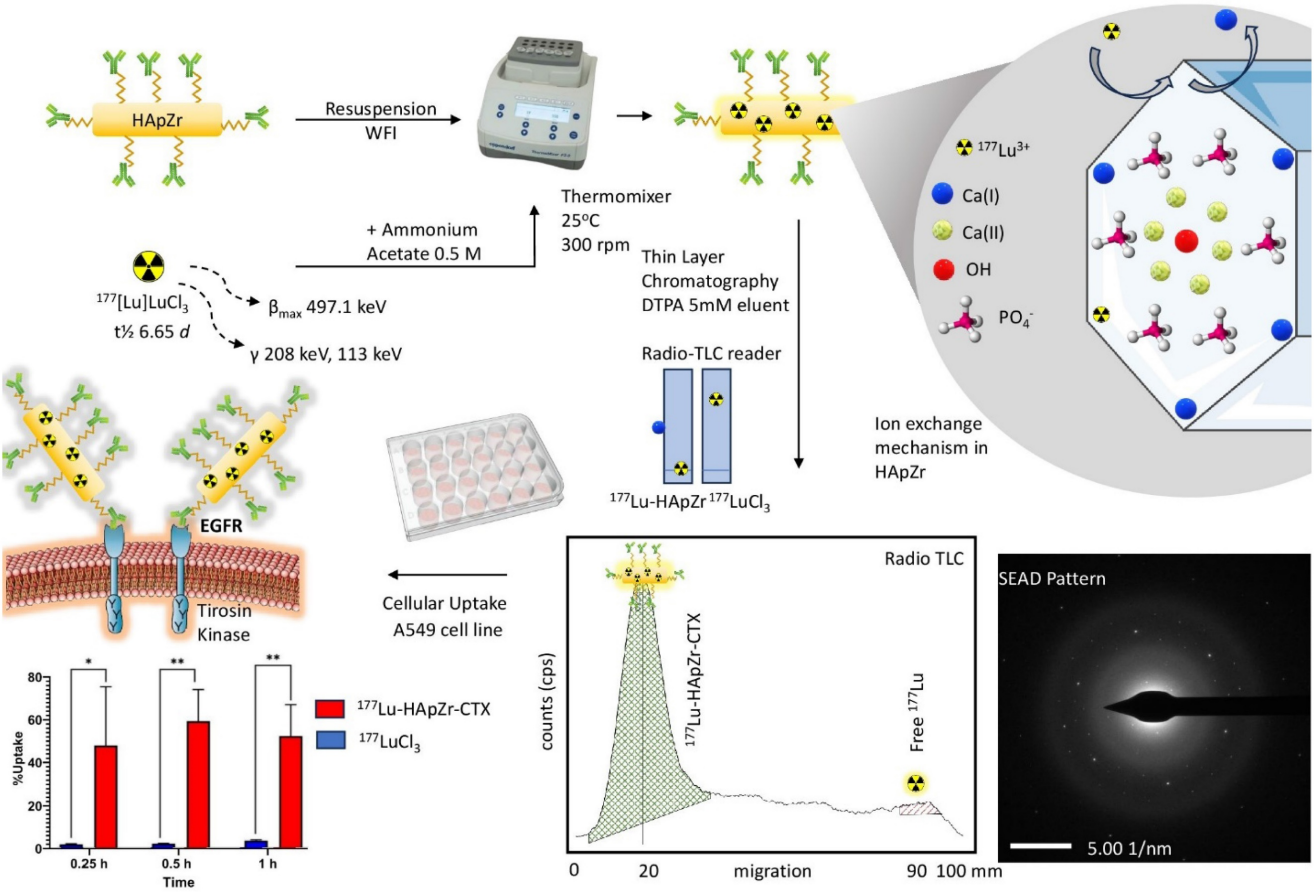
Reaction scheme for loading Lutetium-177 into HApZr-CTX nanoparticle. Radio-TLC was used to identify the radiochemical yield of [^177^Lu]Lu-HApZr-CTX. The cellular uptake study used the A549 cell line at three different time points. ^177^Lu^3+^ was incorporated into HApZr crystal through an ion exchange mechanism in the Ca(I) position.

Radiolabeling of HApZr nanoparticle with ^177^Lu was carried out using the ion exchange principle. ^177^Lu cation will replace the calcium position in the HApZr crystal. This process is quite simple but yields a relatively high radiochemical purity. About 30 minutes of gentle mixing at room temperature was suitable for HApZr-CTX radiolabeling with ^177^Lu. A thin-layer chromatography system consisting of Whatmann 31 ET paper (stationary phase) and 5 mM DTPA (mobile phase) was used to separate labeled nanoparticle and unlabeled/free ^177^Lu. Labeled nanoparticle were heavy enough to be retained at the origin spot, while free ^177^Lu was chelated by DTPA and moved into the solvent front, as shown in Figure 3. The radiolabeled nanoparticle were stable with more than 95% radiochemical purity for 5 days of storage at 10^°^C.

### Cellular uptake study

[^177^Lu]Lu-HApZr-CTX uptake was evaluated on A549 cancer cell lines. The comparison of ^177^Lu as control was carried out in three different time points namely 0.25 h, 0.5 h, and 1 h post-incubation, as shown in Figure 3. The study at 0.25 h showed that the uptake of [^177^Lu]Lu-HApZr-CTX was significantly higher at 40.75 ± 23.39 % compared to ^177^Lu at 1.58 ± 0.24 % (*p*<0.05). At the 0.5 h evaluation, the cellular uptake of [^177^Lu]Lu-HApZr-CTX was 50.44 ± 12.57 %, which decreased to 44.55 ± 12.46 % after 1 h post-incubation. Meanwhile, the cellular uptake of ^177^Lu for 0.5 h and 1 h post incubation was insignificant, with values of 1.83 ± 0.16 % and 3.06 ± 0.34 %, respectively.

### Cellular imaging of HApZr-CTX-FITC

**Figure 4:**
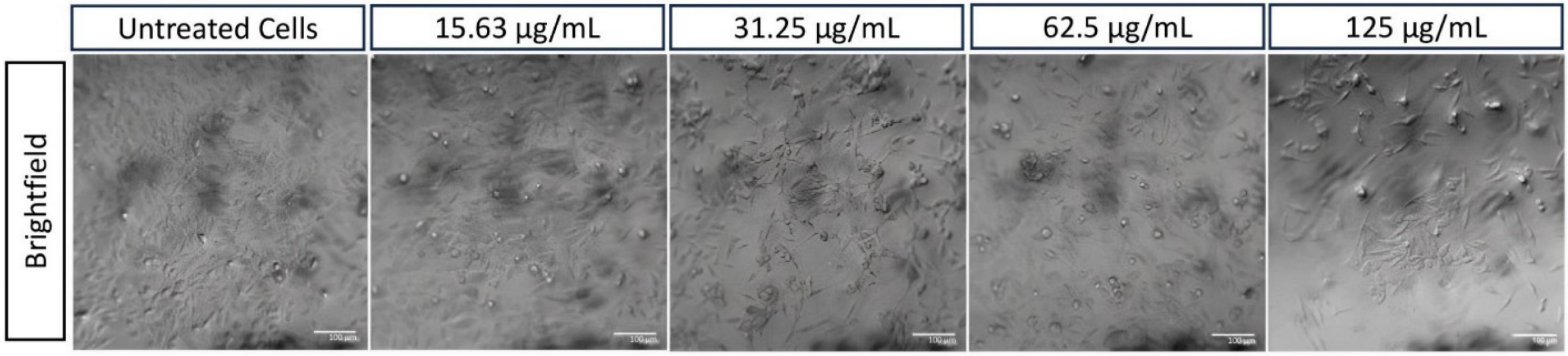
A549 cell morphology after therapy using various concentrations of HApZr-CTX compared with untreated cells as a control condition. Scale bar: 100 µm

**Figure 5:**
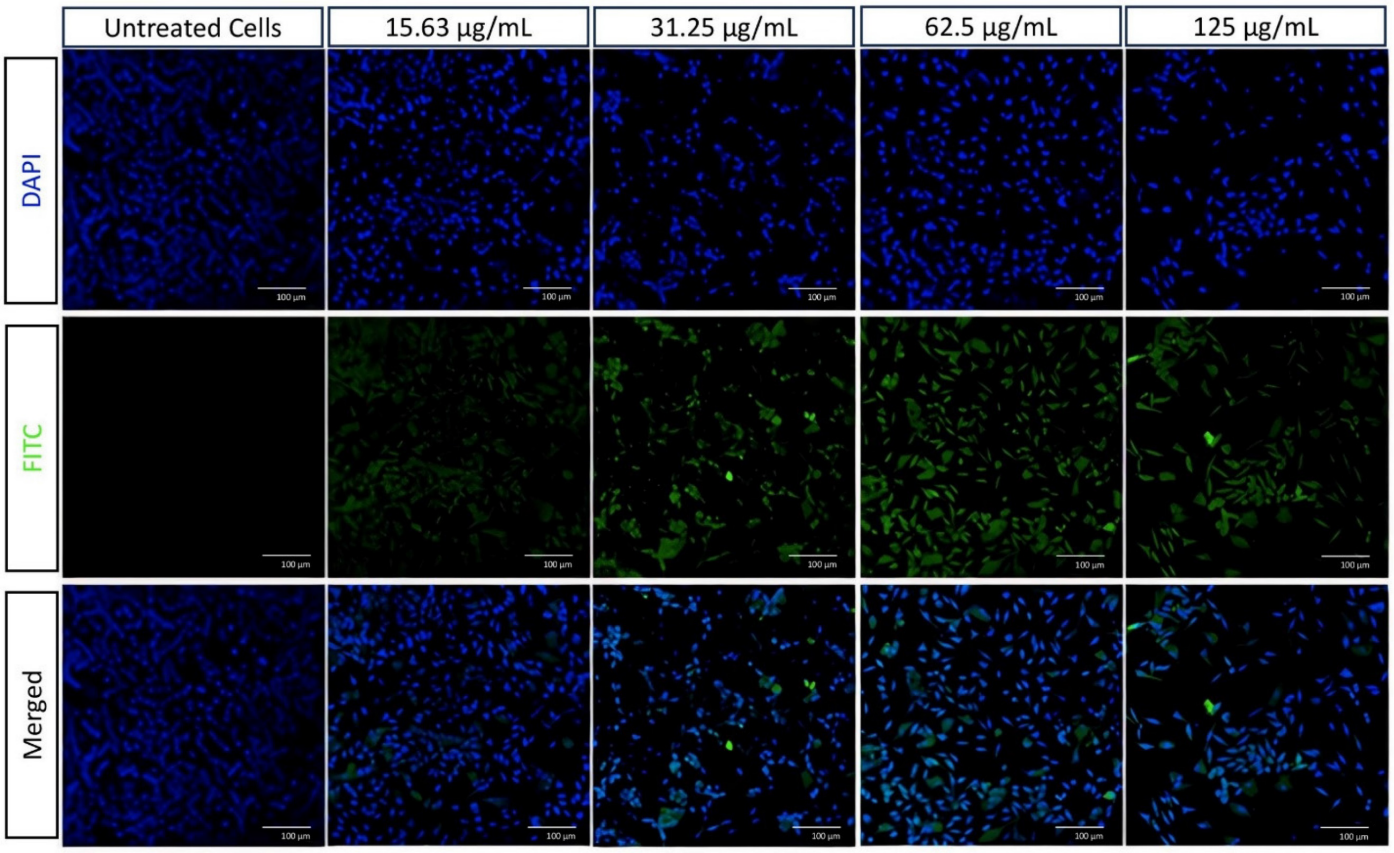
Cellular imaging of A549 cells using CLSM stained with DAPI (blue) and FITC conjugated in HApZr-CTX (green) in various concentrations compared to untreated cells as a control condition. Scale bar: 100 µm

HApZr-CTX showed a dose-dependent cytotoxic effect on A549 cells (Figures 4 and 5) and based on cytotoxicity studies in Figure 7. Cell viability decreased from 31.25 µg/mL to 125 µg/mL based on cellular imaging evaluations.

The internalization of HApZr-CTX was observed by conjugating with FITC. The results confirmed that HApZr-CTX internalized into the cytoplasm of the A549 cells in various concentrations. The cells also showed visible morphological alterations, including changes in cell shape, size, and other visible characteristics.

### ROS generation study

**Figure 6:**
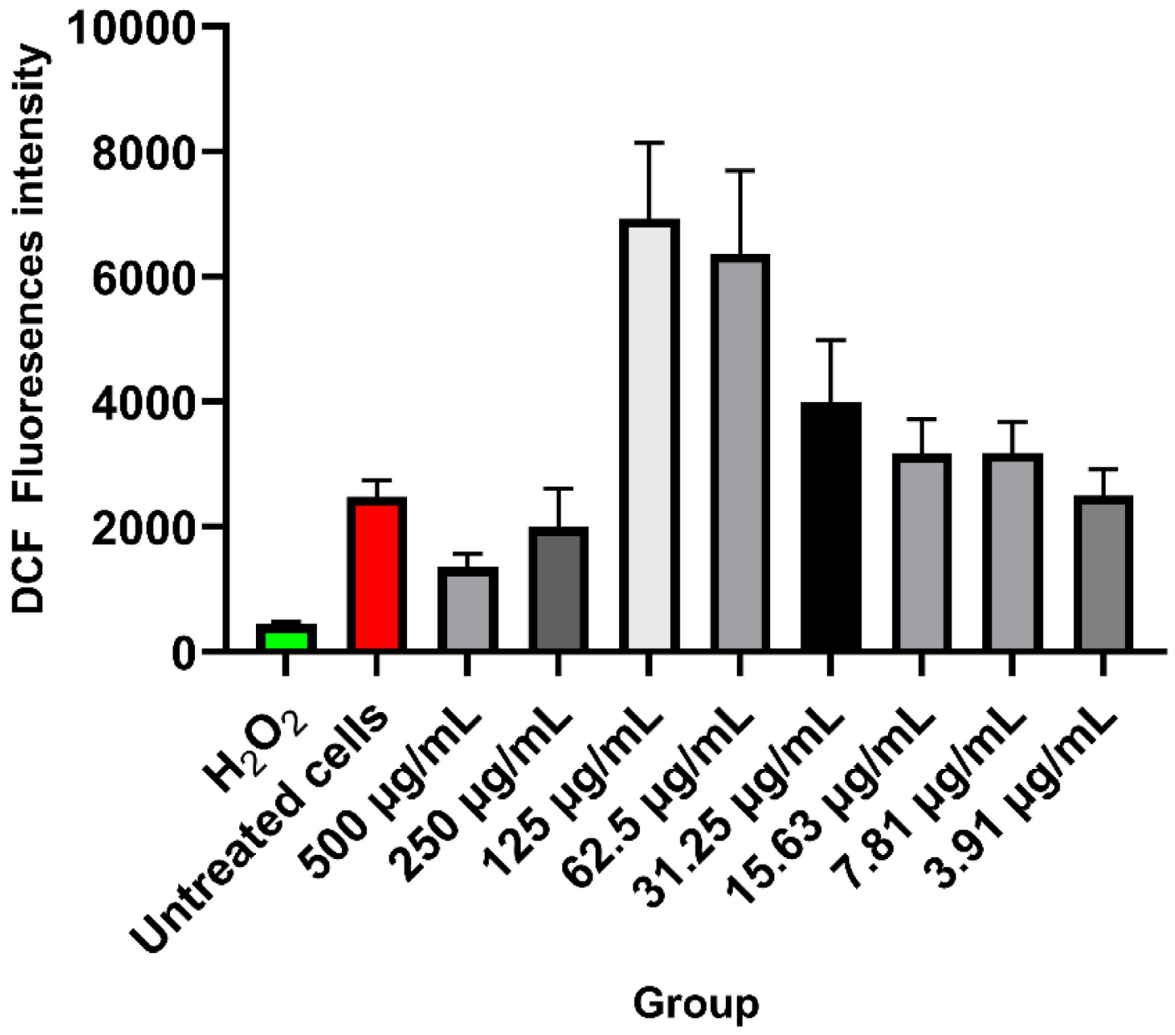
DCF fluorescence intensity of HApZr-CTX in A549 cancer cell line after 24 h post 5 Gy X-ray irradiation (n=4). The variant concentration of HApZr-CTX and Hydrogen Peroxide (H_2_O_2_) as positive control were presented for ROS generation evaluation.

Figure 6 shows the ROS level for each treatment group. DCF fluorescence significantly increased as an indicator for ROS at 125 µg/mL and 62.5 µg/mL of HApZr-CTX nanoparticle concentrations after 5 Gy irradiation. The untreated cells that only received irradiation doses showed a lower level of DCF fluorescence. Higher concentrations at 500 µg/mL and 250 µg/mL also represent lower DCF fluorescence levels than other concentrations.

### Cytotoxic studies of X-ray dynamic therapy

**Figure 7:**
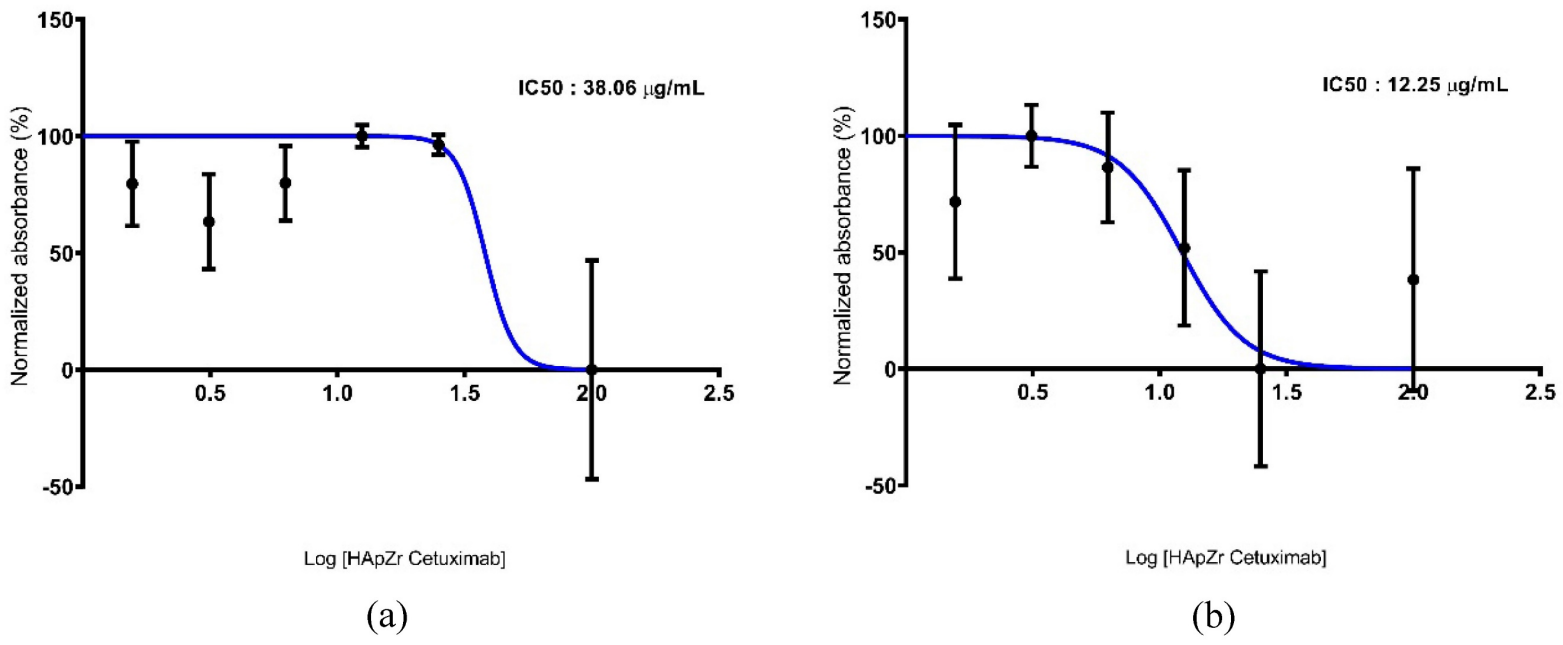
Cytotoxicity studies of HApZr-CTX in A549 cell line. (a) Nonirradiated group; (b) irradiated group.

Figure 7 shows the viability profile of the A549 cell line after therapy using HApZr-CTX with and without irradiation. The results indicate that HApZr-CTX has a cytotoxic effect in a single therapy, with an IC_50_ value of 38.06 µg/mL. Combination with photon irradiation showed increased cytotoxic effects, indicated by a decrease in the IC_50_ value at 12.25 µg/mL.

### Biodistribution study of [^177^Lu]Lu-HApZr-CTX

**Figure 8:**
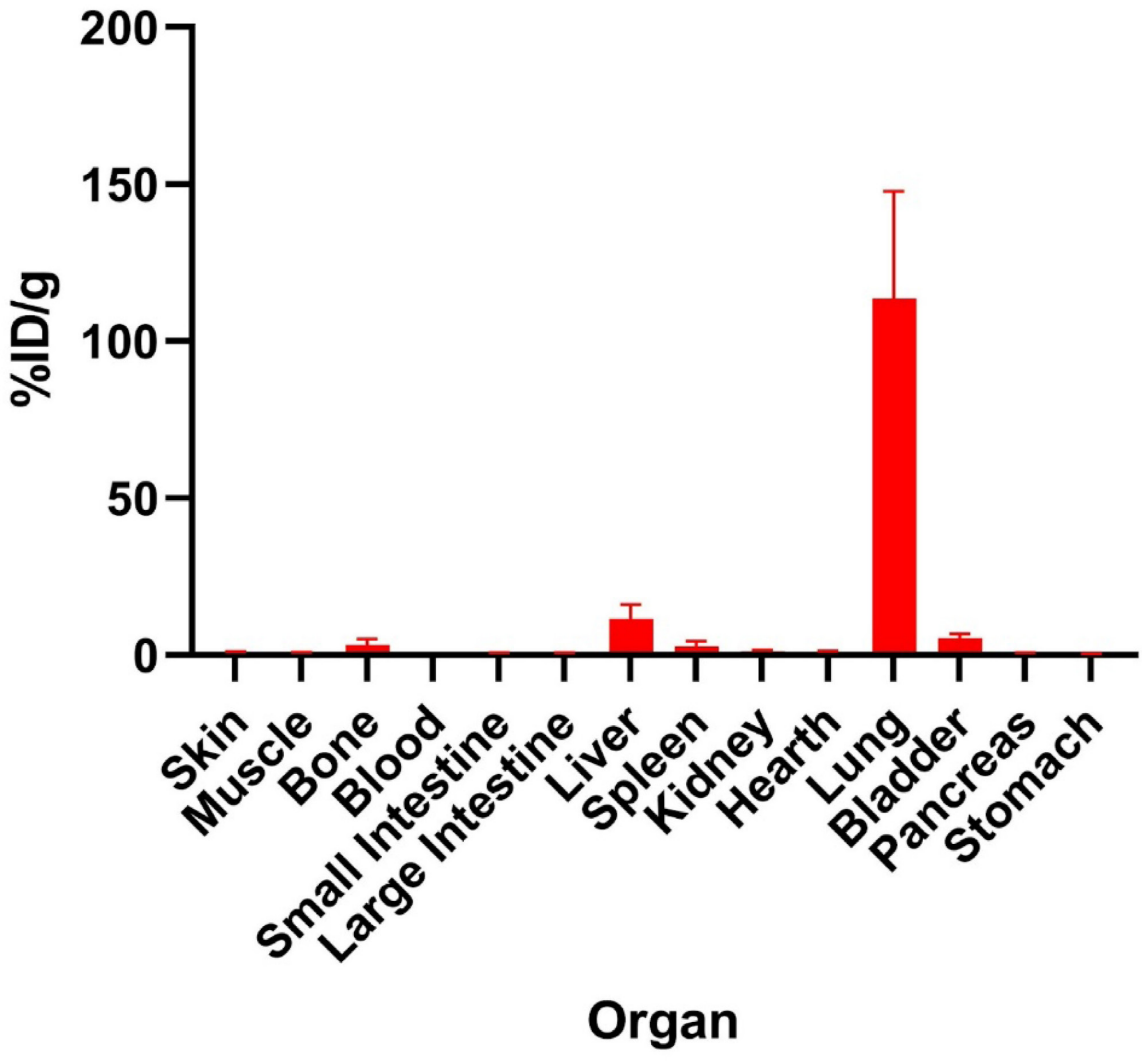
The *ex vivo* biodistribution of [^177^Lu]Lu-HApZr-CTX at 1 h post-injection. Uptake expressed as a percentage of injected activity dose per gram of tissue (%ID/g) in male BALB/c mice injected with 1.25 MBq/100 µL of [^177^Lu]Lu-HApZr-CTX (n=3). The error bar represents the standard deviation (SD).

The *ex-vivo* biodistribution of [^177^Lu]Lu-HApZr-CTX is shown in Figure 8. Specific accumulation in the lungs at 1 h after injection was observed compared to other organs such as the liver and spleen.

## Discussion

Targeted therapy has attracted significant interest in cancer therapy. The use of radionuclide therapy through therapeutic radionuclides showed positive results for clinical applications such as iodine-131 (^131^I) for thyroid carcinoma, [^131^I]MIBG for malignant and metastatic pheochromocytoma, yttrium-90 (^90^Y) anti CD20 immunoglobulin for metastatic neuroendocrine tumors, and [^177^Lu]Lu-PSMA for prostate cancer therapy with high PSMA expression 24.

The combination of radionuclide therapy with an antibody as a carrier to reduce the off-target and specify the molecule to certain receptors has also been widely studied 9,25,26. In this study, CTX, a specific antibody drug for cancer that binds specifically to EGFR on the surface of cancer cells, was used to develop a radioimmunotherapy candidate.

Rather than using a sole antibody, conjugation of nanoparticle with an antibody could enhance the effectiveness of radioimmunotherapy for increasing drug concentration intracellularly through receptor-mediated endocytosis and accumulation in cells. This method is also characterized by improved specificity and avidity of the targeting system, which leads to better therapeutic and diagnostic outcomes, as well as specificity and functionality. Furthermore, it has a higher payload capacity which allows for the delivery of large-scale therapeutic agents, and enhanced immune response to weak antigens, offering benefits to disorders mediated by an unwanted immune response 27,28.

The main consideration of using only antibodies without nanoparticle is the potential health risks of long blood retention in radioimmunotherapy. Antibodies individually may not have high selectivity, which limits specificity and avidity in the targeting system. Considering antibodies do not have a high payload capacity, this can limit the number of therapeutic agents delivered, potentially causing systemic exposure which then leads to immunogenicity and subsequent clearance 29,30. The problem associated with improper radiation dose into healthy cells caused by slow blood clearance, such as hepatotoxicity, has also become a recent consideration in the application of monoclonal antibodies in radioimmunotherapy 31.

Based on the results of this study, the use of antibody conjugated nanoparticle could accelerate cellular uptake of loaded drug compared to sole antibody, about 40% of [^177^Lu]Lu-HApZr-CTX was internalized after 15 minutes of incubation and increased up to 50% after 30 minutes despite a slight decrease after 60 minutes. On the other hand, other studies have shown that cellular internalization of radiolabeled monoclonal antibodies required prolonged incubation. For example, anti-EGFR monoclonal was observed to internalize into the A549 cells, reaching a maximum of 65.8% after 20 minutes and remaining saturated for 60 minutes 32. Meanwhile, [^177^Lu]Lu-DOTA-rituximab and [^177^Lu]Lu-DOTA-trastuzumab needed up to 120 minutes to reach around 35% of cellular uptake 33.

Aside from using radionuclide therapy for cancer, another well-known method that became a standard therapy was using external beam radiation 34. The use of radioenhancers in radiotherapy has also reportedly entered the clinical trial stage 35-37. In this study, cellular staining using DAPI and FITC labeled HApZr-CTX showed internalization in the cytoplasm of A549 cells. On the other hand, HApZr-CTX also showed potential as an anticancer in a dose-dependent manner (Figures 4 and 7b). This cytotoxic activity is derived from the characteristics of HApZr 5. FITC was used as a green fluorescence imaging to observe HAPZr-CTX since the compound did not emit sufficient fluorescence signal. Based on the observations, HAPZr-CTX conjugated with FITC was localized to the cell membrane and cytoplasm. The ligand used was CTX, an anti-EGFR receptor that specifically targets EGFR receptor on the cell membrane surface. A limitation of this study is the absence of membrane staining, which would have provided clearer visualization of the cell compartments.

Cellular ROS generation was detected in the A549 cell line after being treated with HApZr-CTX and irradiated with 5 Gy of X-ray. DCF fluorescence signal indicates cellular ROS generation was gradually increased with the increment of HApZr-CTX concentration and reached the peak at 125 µg/mL. DCF signal dropped drastically when HApZr-CTX concentration doubled to 250 µg/mL and 500 µg/mL. This phenomenon is likely related to nanoparticle quenching of fluorescence signals. Carbon nanoparticle are known to absorb light, including fluorescence signals from DCF probes 38. HApNPs also have fluorescence quenching ability, according to a study by Zhang 39. The decrease in IC_50_ value of HApZr-CTX after irradiation compared to the non-irradiated group indicates synergism between irradiation and HApZr-CTX administration in killing cancer cells. This was in accordance with the ROS results, which showed an increase along with higher HApZr-CTX administration. Therefore, zirconium incorporated into HApZr nanoparticles and modified with CTX as a targeting agent has the potential to be a radioenhancer agent in XDT due to its cytotoxic and ROS generation properties. Using DCF fluorescence signals to evaluate ROS activity has limitations, particularly in terms of specificity. Potential competition with endogenous antioxidants is considered a limitation of this study 40. This could be improved by combining cellular imaging techniques for ROS production. An effective time frame is also important for applying XDT to ensure the radioenhancer already accumulates around the tumor tissue 41, which was not performed in this study.

The functionalized HApZr-CTX nanoparticle also enabled chelator-free labeling of ^177^Lu. The selection of a chelator for radiolabeling is usually complicated because the necessity of the radiochemical and biological characteristics of the produced radiolabeled antibody can be greatly impacted by the chelator choice. Several studies have assessed the possible impact of various chelating agents on radiolabeled monoclonal antibodies that bind to the target receptor 42-44. The radiolabeled immunoconjugate important radiochemical and biological properties may change due to structural modifications caused by the conjugation of an antibody with a chelator and the ensuing radiolabeling. The primary features that have been investigated include the impact of a specific chelator on the final complex formation and stability, as well as any potential chelator-type effects on the distribution of radiolabeled antibodies to organs, tissues, and cancers 45.

Using nanoparticle would change the nature of the whole compound instead of only utilizing the guiding ability of monoclonal antibodies. The intracellular internalization mechanism for radiolabeled functionalized nanoparticle internalization will be different compared to monoclonal antibodies 46. In this study, the *ex-vivo* biodistribution of [^177^Lu]Lu-HApZr-CTX showed high uptake and fast transportation of ^177^Lu into lung organs in normal mice. The uptake was due to enhanced permeability and retention effect instead of active targeting through the binding of CTX into EGFR receptor.

A high uptake was found in lung of normal mice for RIT using [^177^Lu]Lu-HApZr-CTX. This is because EGFR is also expressed in the normal lung even though at a low level 47. EGFR mainly contributes to cell proliferation and lung development. Accumulation in lung is also due to the size of HApZr-CTX. However, the low uptake in the non-target organ becomes an advantage, suggesting that the size and shape of RIT compound contribute to passive targeting, as discussed in previous literature 48. This study had a limitation in evaluating suitable cancer animal models for XDT and RIT. Further evaluation using lung cancer models should be carried out by designing a suitable design, especially for determining the type of cancer model, whether orthotopic or subcutaneous. Modifying HApZr with CTX is expected to increase specificity in lung cancer with low accumulation in normal lung tissue.

## Conclusion

In conclusion, this study examined the dual application of CTX-conjugated HApZr nanoparticles for X-ray dynamic therapy and ^177^Lu-based radioimmunotherapy in lung cancer therapy, which shows potential as a nano drug delivery system and dual-mode targeted therapy simultaneously in one platform. Through *in vitro* assessments, this study observed the internalization of the nanoparticle in lung cancer cells. Substantial accumulation was observed in the lungs of normal mice, with minimal retention in non-target organs. *In vitro* XDT study showed Zr carried by HApZr-CTX could enhance radiotherapy output, which would allow a lower radiation dose of radiotherapy in the future and increase the specificity to minimize the adverse effects. Future assessments are needed to determine dosimetry calculation for effective and safe delivery of radiation in external and internal modes through XDT and targeted radionuclide therapy, respectively.

## Abbreviations

HAp: Hydroxyapatite
NP: Nanoparticle
CTX: Cetuximab
XRD: X-ray powder diffraction
TEM: Transmission Electron Microscopy
DLS-PSA: Dynamic Light Scattering Particle Size Analyzer
XDT: X-Ray Dynamic Therapy
ROS: Reactive Oxygen Species
EGFR: Epidermal Rrowth Factor Receptor
RIT: Radioimmunotherapy
FITC: Fluorescein isothiocyanate
